# PhenoDB: A New Web-Based Tool for the Collection, Storage, and Analysis of Phenotypic Features

**DOI:** 10.1002/humu.22283

**Published:** 2013-02-01

**Authors:** Ada Hamosh, Nara Sobreira, Julie Hoover-Fong, V Reid Sutton, Corinne Boehm, François Schiettecatte, David Valle

**Affiliations:** 1 McKusick-Nathans Institute of Genetic Medicine, Johns Hopkins UniversityBaltimore, Maryland; 2 Department of Molecular & Human Genetics, Baylor College of MedicineHouston, Texas; 3 FS ConsultingSalem, Massachusetts

**Keywords:** phenotyping, mendelian disorders, database, bioinformatics

## Abstract

To interpret whole exome/genome sequence data for clinical and research purposes, comprehensive phenotypic information, knowledge of pedigree structure, and results of previous clinical testing are essential. With these requirements in mind and to meet the needs of the Centers for Mendelian Genomics project, we have developed PhenoDB (http://phenodb.net), a secure, Web-based portal for entry, storage, and analysis of phenotypic and other clinical information. The phenotypic features are organized hierarchically according to the major headings and subheadings of the Online Mendelian Inheritance in Man (OMIM®) clinical synopses, with further subdivisions according to structure and function. Every string allows for a free-text entry. All of the approximately 2,900 features use the preferred term from Elements of Morphology and are fully searchable and mapped to the Human Phenotype Ontology and Elements of Morphology. The PhenoDB allows for ascertainment of relevant information from a case in a family or cohort, which is then searchable by family, OMIM number, phenotypic feature, mode of inheritance, genes screened, and so on. The database can also be used to format phenotypic data for submission to dbGaP for appropriately consented individuals. PhenoDB was built using Django, an open source Web development tool, and is freely available through the Johns Hopkins McKusick-Nathans Institute of Genetic Medicine (http://phenodb.net).

## Introduction

Many databases with overlapping features have been created to record phenotypic aspects of disease. Among these, the Human Phenotype Ontology (HPO) (http://www.human-phenotype-ontology.org) [Robinson et al., 2008] is derived from the recurrent terms in the Online Mendelian Inheritance in Man (OMIM®) clinical synopses and now includes >10,000 defined terms organized into an ontology; the Unified Medical Language System (UMLS) with millions of terms from different sources (http://www.nlm.nih.gov/research/umls); and the Elements of Morphology (http://elementsofmorphology.nih.gov) initiative which describes over 400 features of the face, hands, and feet with definitions and photographs [Carey et al., 2012]. Each of these is tailored for specific purposes, but to our knowledge, there is no extant database that can collect, store, and analyze standardized phenotypic data.

Collection and collation of comprehensive phenotypic information, knowledge of pedigree structure, and clinical testing results are necessary to optimize the application of whole exome and whole genome sequence approaches to explain human phenotypes. Image data (photographs, videos, radiographs, CTs, and MRIs) provide additional valuable information. Mendelian phenotypes often have overlapping, ambiguous, and nonspecific features that challenge precise clinical diagnosis. Ideally, a database to manage this information should facilitate data entry, provide the possibility of links to other systems of phenotypic description, and enable sample tracking and generation of progress summaries.

The underlying purpose of this database was to support the efforts of the NHGRI/NHLBI funded Centers for Mendelian Genomics (CMGs) to find the genes responsible for unsolved Mendelian disorders [Bamshad et al., 2012]. Because the CMGs will receive submissions from a wide variety of healthcare providers from around the world, an initial step in the pipeline involves evaluation of submitted cases regarding suitability for this research project. To facilitate this activity, the database must collate the submitted information in a format that can be easily and reproducibly evaluated and accessed by reviewers in disparate locations. Considering these requirements and the features of the existing tools, we elected to develop a new, robust, comprehensive, and interactive database that would meet these needs.

To this end, we developed PhenoDB, described in detail in what follows. Based on our initial 9 months of usage with more than 572 family and five cohort entries, we find that it is an efficient and useful tool for collection, storage, and analysis of phenotypic information, and we expect that it will have applications beyond its original intended use. Accordingly, we have made it freely available to all. Furthermore, because the phenotypic feature list is standardized and fully searchable, it has the potential to be incorporated into the electronic health record in ways that will revolutionize the integration of genetic information into medicine and public health.

## Database Information

### Overview

**Figure 1:**
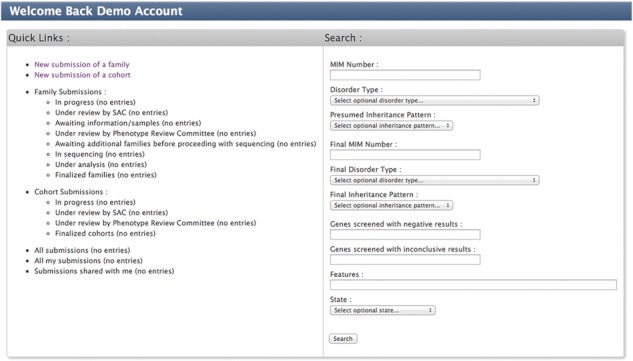
The initial page after login as a submitter. New submission has been highlighted to show the next screen. This page also shows all the programmed searches.

PhenoDB assumes the family as the smallest unit of analysis, whether this is a single affected individual or a multiplex family. It also accepts cohorts for consideration, but additional details of subjects chosen for sequencing (from within a cohort) will likely be required. To enter PhenoDB in any capacity, it is necessary to create an account. User authorizations are granted by a system administrator (see below) and are required for access to the database. The database is Web-based and maintains deidentified data on a secure server. Once accessed, the submitter (presumed to be a health care provider or researcher) has the ability to view any of his/her own previously submitted families/cohorts or to submit a new family (Fig. 1). We also added a sample tracking module and an Analysis and Interpretation module to the database. The sample tracking module is useful for the coordinators and is able to integrate with the sequencing laboratory information management system (LIMS). The analysis module incorporates the deliberations and final conclusions of the Analysis and Interpretation Committee, including genes and variants that are likely causative of the disorder under consideration. We structured these data into fields that can generate a report to the submitter and be displayed in summary tables.

### Initial Fields

**Figure 2:**
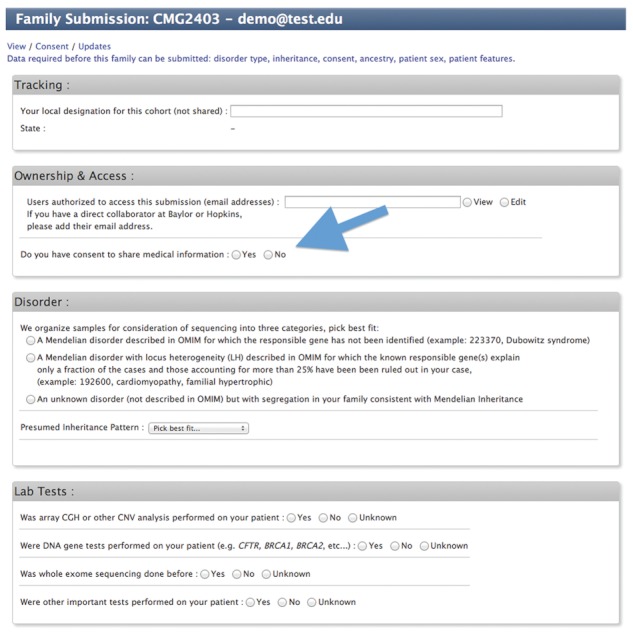
The first page of submission. Please note that a family number has been automatically generated (top of the page) and that the submitter must affirm that consent to share medical information has been obtained.

Electing to submit a new family automatically generates a unique identifier for the family and for members of that family. The submitter is permitted a local designation for a family. This is visible only to the submitter. The submitter may also grant access to other users (who must have an account in the database) for access to a family he/she submits to the database. These other users may have either view only or edit privileges assigned by the primary submitter (Fig. 2).

### Type of Disorder

For the CMG project, we divided disorders into three types: (1) a known OMIM disorder whose causal gene has not yet been identified, (2) a known OMIM disorder with locus heterogeneity, in which the patient in question has been tested for all the genes which each account for more than 25% of cases, and (3) a completely novel disorder not yet described in OMIM. For group 1 disorders, the MIM number and title is used to classify the disorder. For group 2 disorders, the lowest MIM number in the series, followed by the letters LH (for Locus Heterogeneity) is used. The disorder name is the title of the MIM series. For group 3 disorders, the database creates a 700,000 series number that ends with the family number, also created by the database, followed by the letter U (for Unknown). The submitter must put a descriptive label on the family.

### Presumed Inheritance

Because the purpose of the submission is to obtain whole exome sequencing, inheritance excludes mitochondrial inheritance but includes autosomal dominant, autosomal recessive, X-linked dominant, X-linked recessive, Y-linked, isolated cases, and unknown inheritance.

### Consent to Share Medical Information

Although only deidentified data are collected, the submitter must acknowledge that he/she has the patient/parent's consent to share medical information. If this question is answered “No”, then the family cannot be submitted for consideration for sequencing. For efficiency, we delay requesting consent for whole exome/genome sequencing until the family is approved, as the work and time required for the submitter to reconsent the family or for the ELSI committee to review extant consents could be considerable.

### Tests on Proband

These are divided into tests of copy number variation (which platform, if performed); single genes tested with negative and inconclusive results, with a separate box for each gene, so that this is searchable; whole exome sequencing (presumably with low coverage depth and certainly no conclusive results); and other important tests. The submitter chooses what data to enter in the “other important tests” category. In each case, the report associated with the results is uploaded to the database (after identifiers are deleted). This can be done directly by the submitter, or the results can be faxed or mailed (a cover sheet with the family number is automatically generated by the database) to the Center, and the Coordinator will upload them to the database. All uploaded files are encrypted.

### Family Structure

**Figure 3:**
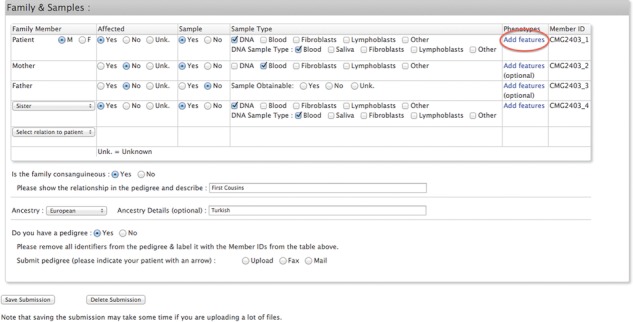
The family information that is collected. An example is prepopulated to show that sample information is also collected. The link to add features (required of all affected individuals in a family) is highlighted.

This section allows entry of relevant family members, defined by their relationship to the proband, as well as sample availability. There is no limit to the number of family members that can be added and any relationship can be represented. Information regarding consanguinity and ancestry (defined by continent of origin but with the ability to give a detailed free-text description) is collected, and a pedigree is requested. We ask that the pedigree be relabeled with the unique, PhenoDB-provided, family, and member number of each individual (Fig. 3).

### Phenotypic Features

**Figure 4:**
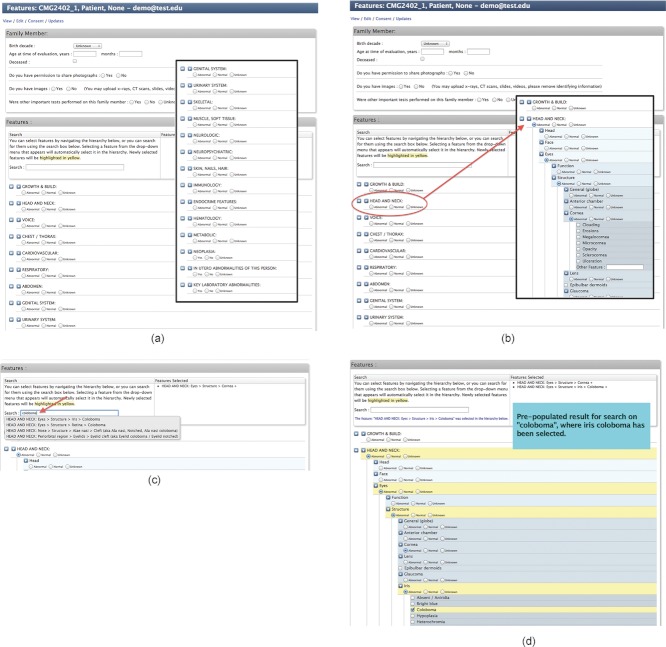
**A:** The individual specific page, including the highest level of the phenotype hierarchy. **B:** An example of feature selection using the hierarchy. Selecting abnormal for any category opens a tree below. **C:** Alternatively the search box can be used to find the desired terms. **D:** Selection of a term automatically selects it (and those higher up) in the hierarchy.

Each individual is entered independently. For every affected individual in the family, phenotypic features are required. The page begins with a request for birth decade rather than birth year (because of US Health Information Portability and Accountability Act (HIPAA) concerns), age when last evaluated, the option to upload photographs (if consented), and other imaging studies, including radiographs, CT scans, MRIs, and videos. Digitized pathology slides can also be loaded in this section (Fig. 4A).

Next is a hierarchical structure of features (Fig. 4A and 4B). The top level consists of 21 categories derived from the OMIM clinical synopses and based upon organ systems. It is expected (and ideal) but not required that each submitter will answer abnormal, normal, or unknown for each of these categories. Selecting abnormal opens the next level in the hierarchy, initially divided into structure and function, and so on. These can be selected down to the level of granularity that is known and/or desired by the submitter. Each string of final features ends with an “Other Feature”: text box to allow for free text entry of features not hard coded into the database. Features entered in these text boxes are reviewed at submission. If the entered features correspond to a standard term, it is corrected; and if it is a new term, the feature is added to a list of potential new features that is reviewed every 3 months for addition of new terms to the database. Terms that appear ≥5 times will be added. This hierarchy includes approximately 2,900 features derived from OMIM and checked against the HPO, Orphanet [Rath et al., 2012], London Dysmorphology Database [Guest et al., 1999], and uses the preferred terms from Elements of Morphology (please see complete list with mappings to the HPO and Elements of Morphology at (http://phenodb.net/help/features) and in Supp. Table S1. Alternatively, a search box can be used to find the desired terms. Entrance of a term automatically selects it (and those higher up) in the hierarchy (Fig. 4C and 4D).

**Figure 5:**
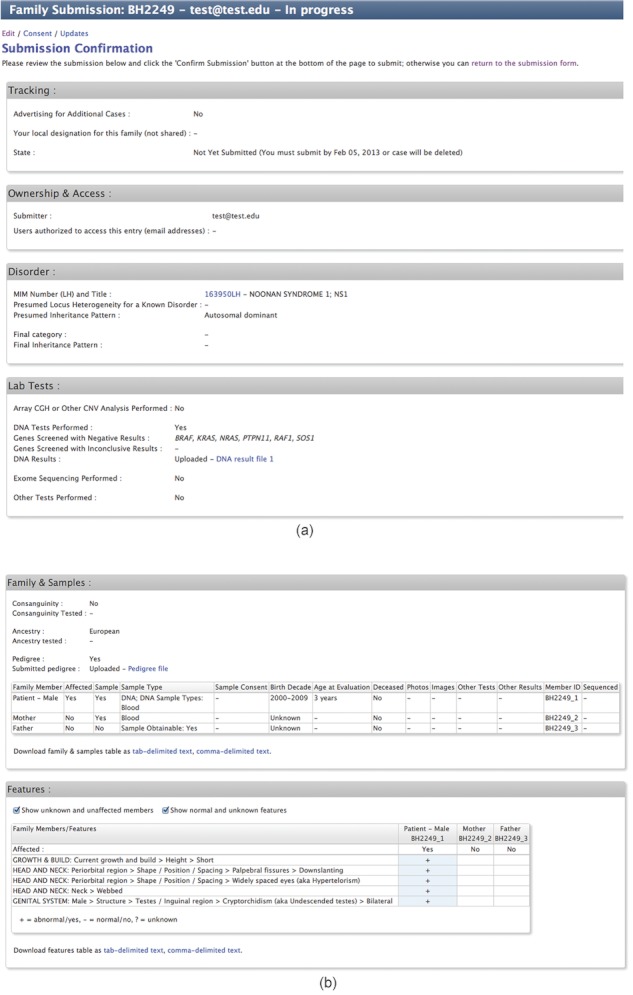
The summary data view for review before submission. This is also the view seen by members of the PRC when reviewing families and by members of the Analysis & Interpretation Committee. A. Top of page. B. Bottom of page.

After completion of phenotypic features for each affected individual, the family is ready to be submitted. Before finalization, the submitter must review and can edit the submission (Fig. 5A and 5B). The tabular view resembles a table in a typical publication. For the CMG project, following finalized submission, no further edits to the family are possible without contacting the coordinator, but this can be changed if the Website is being used for a different purpose.

### User Views

Currently there are several different user types and each has unique views of the data in the database.

- Submitters can always view their own families as well as others for which they have been listed as users. They can edit an entry until submission is finalized.
- *Members of the Phenotype Review Committee* (PRC) have view access to all families and cohorts submitted to the database. The leader has read–write access allowing him/her authority to summarize the deliberations of the PRC for others to view and can change the state (where the submission is in the process) of the family or cohort. Once a family is approved as appropriate for sequencing, the coordinator recontacts the submitter to inform them and to request the consent form used for the family or to help the submitter with reconsenting the patient/family. If the submitter wishes to use his/her own consent (rather than that of the CMG) or has legacy samples from individuals who cannot be reconsented, the coordinator requests that the consents be uploaded for ELSI committee review.
- *Members of the ELSI Committee* can see only the consent forms and none of the family or phenotype information. No one but ELSI committee members and the coordinator can see consent forms. The ELSI committee has its own deliberation box (visible to them) and has back-end-coded data indicating if the subject has consented to submission of data to dbGaP and/or return of results.
- *Members of the Analysis & Interpretation Committee* can view the phenotype and family structure data to guide their analysis. They will deposit their conclusions, which are then displayed in a final table visible to submitters and all others with access.
- *Coordinators* (SAC for Sample Acquisition Coordinator) have full administrative authority to edit fields. Coordinators can delete documents to remove identifiers and reupload them and have their own box for recording discussions with submitters and others.

PhenoDB is fully searchable regardless of the view (i.e., within the restrictions of the user's access). Submitted information is searchable by MIM number (disease), disorder type (1, 2, or 3, see above), presumed inheritance pattern, genes screened, phenotypic features, and results. The return of the search can include a displayed feature summary or not, as desired by the query. This functionality allows for comparison of all individuals present in the database affected with the same disorder or with similar phenotypic features. This information helps the PRC select the families (and individuals within a cohort) most clearly affected with the condition and therefore most likely to result in successful identification of the causative gene. Additionally, a feature summary table including all individuals affected with a particular disease can be directly imported into a manuscript.

### Database Schema

This application is built using Django, a Python based open source Web development tool, and uses MySQL as the underlying database. In addition to the programmed searches, it is easy to query using SQL select commands.

### Future Plans for PhenoDB

We intend to add a mouse over definition (derived from HPO and/or medical dictionaries) for each term in the phenotypic features list as well as links to the Elements of Morphology for a photograph. This will help submitters to pick the correct term if they are not familiar with these and will serve as an educational tool.

Those wishing to use PhenoDB for independent projects and/or laboratories will be able to adapt it for their own purposes and can elect to ignore any module that they do not need. We expect quarterly updates to PhenoDB throughout 2013 and possibly into the future. All updates will be available and versioned at (http://phenodb.net).

## Conclusions

PhenoDB is a robust, useful database for collection, storage, and analysis of phenotypic data, especially in the context of whole exome/genome sequencing approaches to identify the responsible gene and variant. We developed it for the CMG project, an NHGRI/NHLBI funded initiative to ascertain the causal gene for unsolved Mendelian disorders. The utility of PhenoDB extends beyond this initial intent and is likely to benefit any laboratory undertaking clinically relevant whole exome/genome sequencing. We have made the database freely available for download after registration (http://phenodb.net/downloads).

## Supplementary material

Disclaimer: Supplementary materials have been peer-reviewed but not copyedited.
